# The Effect of a Combined Exercise Program on Postural Control and Fine Motor Skills in Parkinson’s Disease: Study Design

**DOI:** 10.3390/ijerph192215216

**Published:** 2022-11-18

**Authors:** Marianna De Maio, Loriana Castellani, Lucia Cugusi, Cristina Cortis, Andrea Fusco

**Affiliations:** 1 Department of Human Sciences, Society and Health, University of Cassino and Lazio Meridionale, Viale dell’Università, 03043 Cassino, Italy; 2 Department of Biomedical Sciences, University of Sassari, Viale San Pietro 43/B, 07100 Sassari, Italy

**Keywords:** Parkinson’s disease, training program, fine motor skills, postural control, grooved pegboard, wobble board

## Abstract

Parkinson’s disease (PD) is a progressive and neurodegenerative disorder defined by physical symptoms such as hand disability and postural instability. To counteract the detrimental effects of PD, physical activity programs showed improvements in overall aspects of physical functioning. Therefore, this protocol will aim to evaluate the effect a of postural and fine motor skills training program in older adults with PD. PD individuals, with mild to moderate stage PD, aged between 65 to 80 years, will be voluntary selected from the Nursing Home Residences and Rehabilitation Centers. Subsequently, they will be randomly assigned to intervention group (PD) to receive a combined training program (postural control and fine motor skills exercises) or to the Control group (CON) to receive a stretching program. Before (PRE) and after (POST) a 12-week program both groups will perform wobble board (WB) and grooved pegboard (GPT) tests. Different performances between groups will be expected: (1) no significant differences between PD and CON group for WB and GPT test values before the beginning of the training intervention (PRE); (2) significantly better WB and GPT test values in PD subjects after the training intervention (POST) when compared to the base values (PRE); and (3) no significant differences in WB and GPT test values in CON subjects after the training intervention (POST) when compared to the base values (PRE). The findings of the present study protocol could be used for future studies investigating clinical populations, such as PD, and the effects of different rehabilitative interventions aiming to improve postural control and fine motor skills performances assessed by WB and GPT tests.

## 1. Introduction

Aging is defined as a natural, continuous and irreversible process that leads to both cognitive and physical decline, characterized by a reduction in coordination, loss of balance and the onset of several diseases [1], such as Parkinson disease (PD). PD is a slow-progression neurodegenerative disease with a high incidence in aged people, affecting 1% to 2% (1.1 million) of the older population above 65 years of age [2,3]. However, definitive conclusions about the etiology of PD have not been reached. It is generally considered to be a consequence of the simultaneous action of toxic and genetic agents (oxidative stress, mitochondrial abnormalities, excitotoxicity, inflammatory factors, environmental neurotoxins, genetic factors and brain aging) able to degenerate the actions of the dopamine neuron in individuals [4,5]. PD is mainly characterized by non-motor and motor symptoms. Non-motor symptoms, such as impairments in memory, communication, visuo-spatial skills, emotional difficulties, anxiety and depression, compromise cognitive abilities, emotionality and personality. Motor impairments, such as tremor disorder and postural changes, result in an increase of rate of falls and dysfunction in ambulation and the fine motor skills system [5,6], thus negatively affecting the individual’s self-care activities and quality of life [7]. Therefore, impairments in these abilities could negatively influence daily life activities and health-related quality of life over the lifespan, especially in a pathological population such as PD subjects.

The evaluation of postural control and fine motor skills provides essential information about neuromuscular and motor coordination systems, particularly when neurodegenerative diseases are involved. Regarding postural control, several assessments are employed. Reaching tests, such as the Star Excursion Balance [8] and the Y Balance tests [9], are the most used due to their reliability and validity in evaluating dynamic postural control. Nevertheless, due to the multifaceted nature of the postural control evaluations, by reflecting the complexity of this ability, new approaches are needed to accurately evaluate dynamic posture. Recently, computerized unstable platforms equipped with triaxial accelerometers, such as Wobble Boards (WBs), have been suggested to be reliable [10] in the evaluation of dynamic balance in different populations using different physical tasks, such as monopodalic or bipodalic stance [11], and with or without visual biofeedback conditions [12]. To evaluate fine motor skills, tests used different tasks such as position changes (sitting or tandem position), time to complete the trial test or the number of transferred blocks during Box and Blocks Test [13], Peg test [14] and the Nine-Hole Peg test [15]. The National Institute of Health Toolbox for the Assessment of Neurological and Behavioral Function [16] indicated the Grooved Pegboard test (GPT) as gold standard because it is able to provide essential and accurate information about manual dexterity in PD [17,18]. Measures extrapolated from postural control and fine motor skills evaluations are used in clinical or field settings to evaluate specific properties in healthy subjects or to highlight the effects of several diseases on these complex abilities and, subsequently, organize individualized training protocols and promote appropriate levels of physical activity among individuals with PD. To increase postural control and fine motor skills, several training programs have been proposed. Studies [19,20,21] showed that postural control exercise protocols, including stepping, walking and/or monopodalic, bipodalic stances, are effective to improve postural control in older individuals with PD. Additionally, fine motor skills interventions (placing beads into a bottle, writing and painting) proved to elicit fine-manual dexterity function in PD patients [22,23].

The Parkinson’s Foundation published the Parkinson’s Exercise Guidelines for People with Parkinson’s recommending, in particular, engagement in functional balance and coordination programs 2–3 days per week in 30–60 min sessions. PD subjects should perform static and dynamic exercises and multi-tasking training with daily integration, always considering safety procedures for subjects’ care [24].

Therefore, the beneficial effects of physical activity programs, in particular combined exercise programs, represent the key factor to manage PD symptoms and delay the pathological and physical decline [25,26], as well as when home confinement is required [27]. Consequently, the aim of the present protocol will be to evaluate the effect of a combined exercise program on postural and fine motor skills using a training program in older adults with PD.

## 2. Materials and Methods

### 2.1. Participants

PD subjects (mild to moderate PD), aged between 65 to 80 years, will be voluntarily selected from Nursing Home Residences and Rehabilitation Centers located in the regional area.

Subjects will be only recruited after medical clearance to exercise, approved by their medical practitioner [28]. The project will be carried-out and presented in the above-mentioned location through a meeting where a detailed explanation of the experimental procedures will be given. Before starting the testing sessions, informed consent from participants or their guardians will be obtained. Subsequently, the Mini Mental State Examination Test Questionnaire for PD [29] will be administered to assess the degree of pathology deterioration. The questionnaire includes seven ordered subsections (orientation, visual registration, attention/mental control, two-set verbal fluency, verbal recall, shifting and concept processing) with a total score of 30 (25–30 normal; 18–24 mild to moderate; <18 severe).

Subjects will be excluded if they: (a) have a score > 3 on the Hoehn and Yahr (H&Y) scale [30]; (b) have visual or musculoskeletal deficits; (c) have dementia or psychiatric abnormalities; (d) participate in any other medical or exercise interventions (additional to the usual received therapeutic treatment) during the study period; (e) be unable to carry out our motor performance tests independently and (f) have a Mini-Mental State Examination for PD score between 25–30 (normal) or <18 points (severe).

### 2.2. Procedures

#### 2.2.1. Preliminary Evaluations

One week before the testing session, subjects’ characteristics will be assessed. Body mass and height will be measured by means of a scale with integrated stadiometer with a precision of 0.1 kg and 0.1 cm (Seca, model 709, Vogel & Halke, Hamburg, Germany), and body mass index (BMI) will be calculated [31]. Waist circumference will be measured at the narrowest part of the abdominal region (if the narrowest part of the abdominal region is not clearly distinguishable, the waist will be measured midway between the 10th rib and the crest of the pelvic bone); hip circumference will be measured horizontally at the most protruding points in back of the gluteal region, side and front. Waist to hip ratio (WHR) will be calculated.

Subsequently, subjects will be recruited for the single-blind study and randomly allocated in two groups: PD group and Control group (CON group). PD group will participate in the combined training program; CON group will participate in a stretching program. Before (PRE) and after (POST) the combined training program (intervention) both groups will perform the tests (described in Section 2.2.3) in a randomized order. Participants in both groups will be instructed to continue with their usual care and advised not to change their daily activities during the trial. All the assessments will be carried out when participants will be in the ‘‘on’’ phase (i.e., when medications will be working and symptoms controlled).

Subjects will be able to withdraw from the study at any time for any reason without any consequences.

#### 2.2.2. Sample Size

For the calculation of sample size, no a priori hypothesis was established due the lack of previous data regarding combined exercise program (postural control and fine moto skills) in subjects with PD. For this reason, a pilot study will be performed to estimate the effect size (ES) of the intervention. The pilot study’s recruitment goal will be 15 subjects with PD for each group. Therefore, the ES, using Cohen’s d, based on the results of the pilot study, will be determined. In line with similar research, an ES value less than 0.2 will considered trivial, from 0.2 to 0.5 small, greater than 0.5 to 0.8 moderate and greater than 0.8 large [32].

#### 2.2.3. PRE and POST Evaluations

During this phase, all the subjects will perform a period of free practice with tests (WB and GPT) and procedures. The following test will be administered:

- Wobble Board

The platform is composed of a wooden circular table (diameter 40 cm, height 2 cm) placed on a hemispherical plastic support (diameter 28 cm, height 6 cm) and equipped with a USB connection cable. Connecting the board to a computer, it will be possible, through a proprietary software, to visualize six concentric circles and a yellow motion marker (MM) displayed in relation to the board tilt grade. The trial countdown, as well as the time spent into target zone (TZ), will be showed on the software screen. The TZ will be indicated by a red circle showing the 0° (±1°) tilt angle measured by the triaxial accelerometer. The evaluation will be performed for both lower and upper limbs.

**Figure 1:**
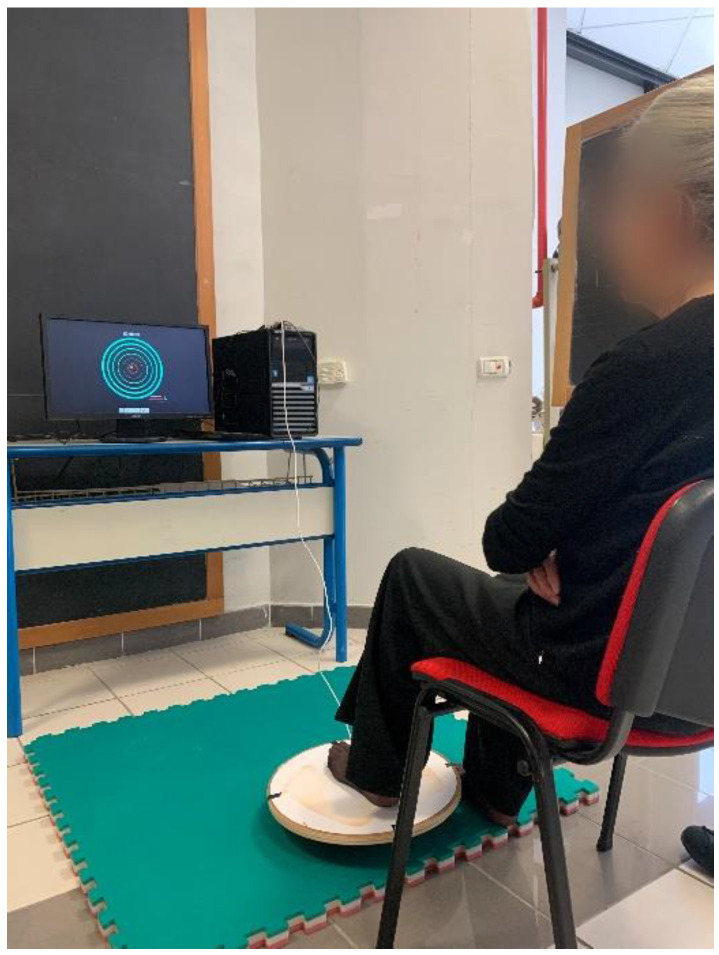
Standard lower limb Wobble Board test position.

The aim will be to keep the MM within the TZ for as long as possible during the recording period. The upper limbs’ test session will consist of a 3-min familiarization on the WB (1 min sitting recovery) followed by three 15-s trials (right and left leg) with 1-min sitting recovery in between. During the test, the TZ will display different motion patterns: clockwise, counterclockwise, antero-posterior, medial-lateral. The lower limbs’ test session will consist of a 3-min familiarization on the WB (1 min sitting recovery) followed by three attempts of 30 s per foot with one minute of rest in between [10]. During lower limb tests, subjects will be required to be in a seated position on a chair with back support with the hands resting on their legs and WB placed in front of the chair (Figure 1).

**Figure 2:**
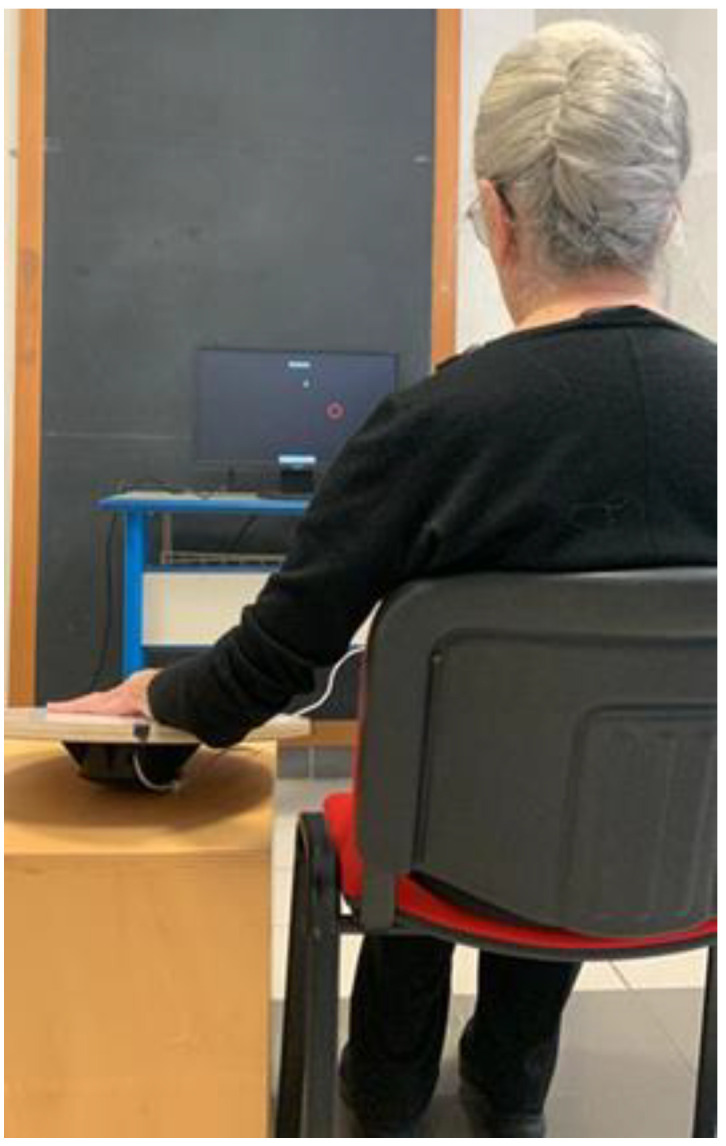
Standard upper limb Wobble Board test position.

During upper limb tests, subjects will be in a seated position, with the tested limb placed at 90° on the WB and the contralateral one (limb not performing the test) resting on the lower limb of the same side. The WB will be placed on a table and the monitor at eye level (Figure 2).

The starting limb and the order of conditions will be randomly chosen.

The test trials will be stopped and repeated if the subjects: (1) use the arms for support; (2) brace the raised leg against the contralateral leg; and (3) drop off the WB.

- Grooved Pegboard Test

**Figure 3:**
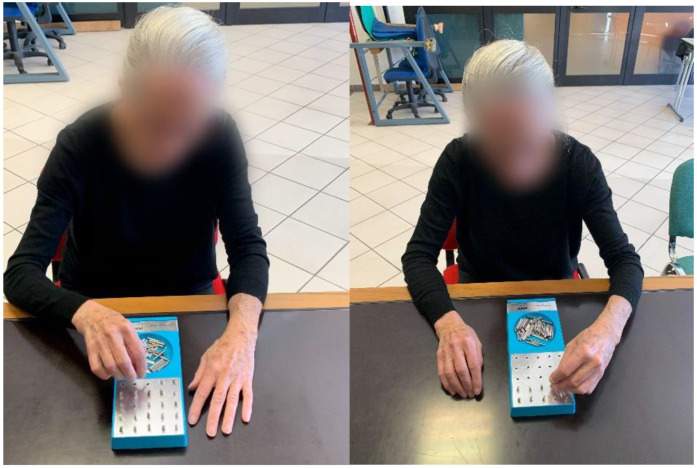
Standard Grooved Pegboard test position.

The GPT (Lafayette Instrument, Lafayette, IN, USA; model 32025) is equipped with a square pegboard (10 cm × 10 cm) with 25 holes arranged in a 5 by 5 configuration, with random keyhole orientation and green steel pegs (diameter = 0.4 cm; model 32104) with a key along one side, located in a spherical tray above the keyholes. After the description of the testing procedures, the subjects will familiarize themselves with the task by filling only the first top row. Subsequently, subjects will be instructed to insert pegs one by one into the pegboard, as fast as possible, completing the rows from left to right for the right limb and from right to left for the left limb, from top to bottom (with 1-min recovery in between). Subjects will be free to perform trials when they prefer [33]. Only the dominant hand will be assessed, and all individuals with PD will perform the GPT two times. The recording time will start when subjects take the first peg and will stop when the last peg is inserted. The time to complete the GPT trials will be recorded by the operator using a digital stopwatch [18]. Lastly, if a peg falls, subjects will have to leave it and continue the test. The GPT will be placed on a table and subjects will be in a comfortable sitting position, with the contralateral limb (limb not performing the test) resting on the table (Figure 3).

#### 2.2.4. Intervention

For 12 weeks, the PD group will receive at least one 60-min session twice a week of a supervised combined training program performed in the participating facilities. The PD group will perform a combined training program (balance and fine motor skills exercises), while the CON group will perform a stretching program. The stretching program will be adopted because of its positive applications in social and emotional aspects, in integrating real tasks from daily life activities and in improving overall physical aspects such as tremor, rigidity, bradykinesia, balance and motor coordination impairments, characterizing PD disease [34].

- PD group

The exercise sessions will include a 10-min warm-up and a cool-down of 5-min of walking, joint mobility (for upper and lower limbs) exercises in clockwise and counterclockwise circling and breathing exercises and followed by 45 min postural control training. After each exercise, subjects will perform a 1-min break in a comfortable seated position.

For postural control training, progression during the intervention period will be reached. For example, visual information will be trained by closing the eyes or looking up, down and sideward; proprioceptive system will be elicited using different unstable surfaces with respect to stable platforms and vestibular system will be disturbed using music or inhibited with appropriate earphones to isolate subjects from the external setting.

- Postural control exercises: bipodalic and monopodalic position (3 sets for 30-s (for limb)); stand up on the feet’ sole (10 repetitions, 3 sets); stand up on heels (10 repetitions, 3 sets); calf raises (10 repetitions, 3 sets); hip abduction (12 repetitions, 3 sets); get up and sit down from a chair (5 repetitions); walking with stop (5 min); walking with change of direction (5 min); walking on a stable surfaces (5 min); walking on an unstable surfaces (5 min); walking with a tennis ball in hands (5 min) and walking and crossing several obstacles (5 min).
- Fine motor skills exercises for lower limbs: in sitting position, lift legs forward by placing a tennis ball between the feet trying not to let it fall (10 repetitions, 3 sets); sitting position and barefoot, grab and drop a towel with toes (for limb) (10 repetitions, 3 sets); pass a tennis ball from foot to foot (20 steps); and in sitting position and barefoot, play a musical carpet (5 min) and toe raises with an elastic band (10 repetitions, 3 sets (for limb)).
- Fine motor skills for upper limbs: paper folding (5 repetitions, 3 sets); play a musical carpet (3 min); create shapes with paper (5 min); finger painting (5 min); trace a drawing (3 min); make bracelets (5 min); count coins and put in a moneybox; close a bottle (10 repetitions, 3 sets); press stamp on paper (10 repetitions, 3 sets); make letters from plasticine (3 min); and press shaped blocks on plasticine (3 min).

- CON group

The exercise programs will include stretching exercises, as follows:

- Stretching during seated position: straight arm forward rotation; straight arm backward rotation; straight arm up and down flapping; straight arm horizontal abduction and adduction; trunk rotation and hold. During standing position (with hands rest on chair back): hamstring stretch; calf stretch; upper trapezius stretch; straight leg forward kick; straight leg backward kick; straight leg hip adduction and abduction; and alternate knee raise (30-s, 2 cycles with 20 s rest in between) [35].

#### 2.2.5. Work Environment and Safety of Procedures

The project will be conducted in the selected Nursing Home Residences and Rehabilitation Centers. To ensure adequate safety standards, the setting designated for the protocol will be equipped with a first aid kit and a semiautomatic defibrillator. Given the progressive cognitive and physical function decline of PD, a neurologist will supervise the study protocol and during each test session, and physical exercise experts will take care of the subjects’ due precautions, such as soft surfaces, harnesses and various aids, to ensure the safety of each PD individual. Moreover, preventive measures will be taken to prevent the risk of Coronavirus disease 2019 (COVID-19) transmission. The designed setting will be periodically sanitized, and testing surfaces will be cleaned with alcohol-based disinfectants before and after each use. Lastly, the use of masks (FFP2) and gloves by the specialists will be mandatory.

#### 2.2.6. Statistical Analysis

To ensure confidentiality and protection of personal data, subjects’ data will be de-identified indicating a numerical code for each participant.

The ‘Intervention’ will be considered as an independent variable, whereas WB and GPT will be considered as dependent variables. The collected data for the analysis of WB and GPT will be expressed in time in seconds (s). Means and SDs will be calculated for each variable. The normal distribution of data will be assessed by the Shapiro–Wilk test. If data are normally distributed, parametric statistical analysis (Analysis of Variance (ANOVA) for repeated measures) will be performed. If data are not normally distributed, non-parametric statistical analysis (Kruskal–Wallis ANOVA for ranked data) will be performed. Therefore, the appropriate analysis will be used to test for differences between PD and CON groups over time (PRE and POST).

In line with previous research, statistical significance will be set at a level of *p* < 0.05. The data will be analyzed using STATA 15 (StataCorp LP, College Station, TX, USA). In addition, the intervention will be considered effective if the evaluated variables (WB and GPT) show improvements in the PD group compared to the CON one.

## 3. Discussion

The purpose of the present study will be to evaluate the effect of a combined training program in older adults with PD. In particular, the proposed 12-week training program will aim to improve postural control and fine motor coordination in mild to moderate PD subjects.

PD is commonly considered as a form of “accelerated aging” of the nervous system affecting subjects’ ability to control movements with tremor disorders [36,37]. The major characteristics of PD are impairments in movement while resting or moving, rigidity [36] and more difficulties in executing simultaneous movements and sequential tasks with respect to simple ones [5]. In particular, the progression of PD leads to a phenomenon called festination, resulting in a decrease in gait speed and an increase in the rate of falls with a consequent loss of independency [5]. Moreover, several studies have reported the negative association between fine motor skills, especially hand disability, and cognitive dysfunction in the PD population [38,39]. In fact, the dopamine neuron depletion caused by PD influences various domains of executive functions, motor coordination and psychomotor speed [40]. These functional disabilities compromise the regular daily activities required for dressing, ascending or descending steps, to self-take care and/or walking. Therefore, the implementation of training interventions to improve postural control and fine motor coordination in PD subjects is fundamental.

Studies proposed several kinds of training programs to improve these abilities in subjects with PD. In the last decade, evidence supported dance activities, aquatic-based exercise and oriental disciplines as effective symptom management modalities. It emerged, in fact, that dance classes could improve functional abilities [41], quality of life [42] and activities of daily living in a PD population [43]. About oriental disciplines, Tai Chi resulted in promising gains in mobility and balance, and it was demonstrated to be safe and popular among subjects with PD at an early stage of the disease [44]. Furthermore, aquatic exercise improves the motor impairments of PD, and it seems to achieve greater benefits than land-based exercise on balance capacity, fear of falling and health-related quality of life in PD populations with a mild to moderate degree of disability [45].

On the other hand, among different single training protocols, combined exercise programs (postural control and fine motor skills exercises) might be more effective. In fact, intervention protocols including several physical activities such as multidirectional stride training, climbing and descending, stretching forward and sideways, obstacles, turning around, stepping, getting up and sitting down, specific trunk rehabilitation exercises, passive spinal joints mobilization, writing, drawing, bouncing a ball during gait, maintaining balance while standing on stable and unstable support bases of different consistency and walking with open or closed eyes are commonly used approaches, which show improvements in muscle activation, fine coordination and postural control and decrease the risk of falls [19,20,21,22,23,46,47].

In line with such evidence, we hypothesized that different performances between groups will expected: (1) no significant differences between PD and CON group for WB and GPT test values before the beginning of the training intervention (PRE-intervention); (2) significantly better WB and GPT test values in PD subjects after the training intervention (POST-intervention) when compared to the base values (PRE-intervention); and (3) no significant differences in WB and GPT test values in CON subjects after the training intervention (POST-intervention) when compared to the base values (PRE-intervention). Therefore, thanks to the future findings of the present study, health professionals could provide individualized training protocols to improve postural control, fine motor skills and perform a regular level of physical activity [24]. Moreover, WB and GPT tests could be practical, inexpensive, administrable and accurate tools to provide essential and useful information about the evaluation of postural control and fine motor skills, especially in light of the progressive degenerative course of PD.

## 4. Conclusions

Cognitive status is strongly associated with postural control and fine motor skills in subjects with PD. With the progression of PD, the most common symptoms are movement-related tremor, rigidity, slowness of movement, postural instability, difficulty with walking and gait and difficulty in motor coordination affecting quality of life. For this reason, training protocols, due their beneficial effects in improving postural control and fine skills, are widely used in PD. Combined postural control and fine motor skills interventions are particularly useful in preventing falls, improving fine coordination and quality of life. In conclusion, the findings of the present study protocol could be used for future comparison studies investigating such a clinical population and the effects of different rehabilitative interventions aiming to improve postural control and fine motor skills performances assessed by WB and GPT tests.
